# Identifying and Interrupting Superspreading Events—Implications for Control of Severe Acute Respiratory Syndrome Coronavirus 2

**DOI:** 10.3201/eid2606.200495

**Published:** 2020-06

**Authors:** Thomas R. Frieden, Christopher T. Lee

**Affiliations:** Resolve to Save Lives, New York, New York, USA

**Keywords:** superspreading events, SSEs, coronavirus, viruses, severe acute respiratory syndrome coronavirus 2, SARS-CoV-2, 2019 novel coronavirus disease, COVID-19, respiratory infections, zoonoses, outbreaks, disease control, pathogen-specific factors, host factors, environmental factors, behavioral factors, response factors

## Abstract

It appears inevitable that severe acute respiratory syndrome coronavirus 2 will continue to spread. Although we still have limited information on the epidemiology of this virus, there have been multiple reports of superspreading events (SSEs), which are associated with both explosive growth early in an outbreak and sustained transmission in later stages. Although SSEs appear to be difficult to predict and therefore difficult to prevent, core public health actions can prevent and reduce the number and impact of SSEs. To prevent and control of SSEs, speed is essential. Prevention and mitigation of SSEs depends, first and foremost, on quickly recognizing and understanding these events, particularly within healthcare settings. Better understanding transmission dynamics associated with SSEs, identifying and mitigating high-risk settings, strict adherence to healthcare infection prevention and control measures, and timely implementation of nonpharmaceutical interventions can help prevent and control severe acute respiratory syndrome coronavirus 2, as well as future infectious disease outbreaks.

Severe acute respiratory syndrome (SARS) coronavirus 2 (SARS-CoV-2) continues to spread (*1*). Although we still have limited information on the epidemiology of coronavirus disease (COVID-19), there have been multiple reports of superspreading events (SSEs) (*2*–*4*). During recent severe outbreaks of SARS, Middle East respiratory syndrome (MERS), and Ebola virus disease, SSEs were associated with explosive growth early in an outbreak and sustained transmission in later stages (*5*–*7*). Here, we review the factors that contribute to SSEs and implications for control of SARS-CoV-2.

SSEs are not limited to emerging infectious diseases. In the early 20th century, Mary Mallon (Typhoid Mary), an asymptomatic typhoid carrier who worked as a cook, infected >50 persons (*8*–*10*). An ingenious and elegant but little-known study of tuberculosis demonstrated that many patients, even those with smear-positive, cavitary tuberculosis, were not highly infectious but that 3 of 77 patients accounted for 73% of the infectious burden (*11*). In 1997, Woolhouse et al. observed that 20% of the population contributed to >80% of transmission and suggested targeting interventions to the core 20% (*12*). SSEs have also caused explosive outbreaks of measles, including among vaccinated persons (*13*).

During the 2003 SARS epidemic in Beijing, China, 1 hospitalized index patient was the source of 4 generations of transmission to 76 patients, visitors, and healthcare workers (*14*). During the MERS outbreak in South Korea, 166 (89%) of 186 confirmed primary cases did not further transmit the disease, but 5 patients led to 154 secondary cases (*15*). The index patient transmitted MERS to 28 other persons, and 3 of these secondary cases infected 84, 23, and 7 persons. During Ebola, SSEs played a key role sustaining the epidemic: 3% of cases were estimated to be responsible for 61% of infections (*6*).

SSEs highlight a major limitation of the concept of R_0_. The basic reproductive number R_0_, when presented as a mean or median value, does not capture the heterogeneity of transmission among infected persons (*16*); 2 pathogens with identical R_0_ estimates may have markedly different patterns of transmission. Furthermore, the goal of a public health response is to drive the reproductive number to a value <1, something that might not be possible in some situations without better prevention, recognition, and response to SSEs. A meta-analysis estimated that the initial median R_0_ for COVID-19 is 2.79 (meaning that 1 infected person will on average infect 2.79 others), although current estimates might be biased because of insufficient data (*17*).

Countermeasures can substantially reduce the reproductive number; on the Diamond Princess cruise ship, an initial estimated R_0_ of 14.8 (≈4 times higher than the R_0_ in the epicenter of the outbreak in Wuhan, China) was reduced to an estimated effective reproductive number of 1.78 after on-board isolation and quarantine measures were implemented (*18*). In Wuhan, aggressive implementation of nonpharmaceutical interventions (NPIs) in the community, including a cordon sanitaire of the city; suspension of public transport, school, and most work; and cancellation of all public events reduced the reproductive number from 3.86 to 0.32 over a 5-week period (C. Wang et al., unpub. data, https://doi.org/10.1101/2020.03.03.20030593). However, these interventions might not be sustainable.

## Drivers of SSEs

Although SSEs appear to be difficult to predict and therefore difficult to prevent, understanding the pathogen, host, environmental, and behavioral drivers of SSEs can inform strategies for SSE prevention and control (*19**,**20*) (Table). The potential impact of these factors has been analyzed (*5*). We summarize the evidence for multiple pathogens to facilitate a more generalized approach that can be applied to the current COVID-19 pandemic.

**Table:** Factors that increase the risk for superspreading events and implications for prevention and control of COVID-19*

Factor	Disease	Epidemiologic role	Implications for control of COVID-19
Pathogen	Tuberculosis	Certain strains of *Mycobacterium tuberculosis* are more infectious, and patients ill with these strains should be prioritized for examination of a larger circle of contacts (*21*,*22*)	Continued monitoring for genetic change and for changes in the epidemiology of transmission
Host	Influenza	Viral shedding and risk for transmission among asymptomatic and presymptomatic persons can result in influenza transmission (*23*), particularly in closed settings with minimal ventilation (H. Nishiura et al., unpub. data, https://doi.org/10.1101/2020.02.28.20029272)	Identification of factors associated with increased transmissibility and rapid intervention to prevent transmission from similar patients prospectively; further characterization of risk for asymptomatic transmission
Environment	SARS	Airborne transmission of SARS can result in environmental spread of disease in community (*24*) and healthcare settings (*25*)	Assess changes in plumbing and ventilation that may be needed to reduce risk for spread; increase social distancing; reduce mass gatherings in closed environments; ensure effective triage, isolation, and general infection control in healthcare facilities
Behavior	Ebola	Inaccurate perceptions of Ebola risk can result in behaviors that increase the probability of transmission (*26*,*27*)	Promote handwashing, cough etiquette, and safer care-seeking behavior, including mask-wearing by persons who are ill, and ensure that timely and accurate messaging about risk and behavioral preventive measures are tailored to and reach affected populations
Response	MERS	Timely implementation of control measures can reduce outbreak duration and number of transmission events (*28*)	Rapidly identify and isolate cases to reduce transmission; implement large-scale NPIs in affected areas within 1 week

*COVID-19, coronavirus disease; MERS, Middle East respiratory syndrome; NPIs, nonpharmaceutical interventions; SARS, severe acute respiratory syndrome.

Pathogen-specific factors include binding sites (*29*), environmental persistence, virulence, and infectious dose. Strains of some organisms might be more readily transmissible than other strains of the same species (*21*,*22*). Mutation can potentially lead to increased infectivity (*6*); one preliminary report suggested that SARS-CoV-2 might have 2 distinct genetic subtypes, with the less lethal form becoming more dominant as a result of treating and isolating infected persons (*30*). Monitoring for genetic adaptation, both by whole-genome sequencing and epidemiologic investigation, will determine whether transmissibility of SARS-CoV-2 is evolving and whether variants of the virus are more readily transmitted.

Host factors include duration of infection (prolonged carriage), location and burden of infection (e.g., laryngeal or cavitary tuberculosis), and symptomatology (e.g., transmission of influenza during the prodromal phase) (*23*). All SARS superspreaders were symptomatic. The potential for and extent of transmission of COVID-19 from asymptomatic infected persons has not yet been fully characterized, although probable asymptomatic transmission has been documented in at least 1 family cluster (*31*). Epidemiologic analysis is required to understand the proportion of COVID-19 transmission which occurs before symptom onset, whether children are effective transmitters, and to identify host factors that might be associated with increased infectivity (*32*).

Environmental factors include population density and the availability and use of infection prevention and control measures in healthcare facilities. SARS and MERS had relatively low rates of person-to-person transmission but caused explosive outbreaks in healthcare settings (*28*). Rapid person-to-person transmission of COVID-19 appears likely to have occurred in healthcare settings, on a cruise ship, and in a church (*3*). In a study of 110 case-patients from 11 clusters in Japan, all clusters were associated with closed environments, including fitness centers, shared eating environments, and hospitals; the odds for transmission from a primary case-patient were 18.7 times higher than in open-air environments (H. Nishiura et al., unpub. data, https://doi.org/10.1101/2020.02.28.20029272). SARS-CoV-2 is present in stool (*33*); ensuring cleanliness of toilets and other potentially contaminated surfaces is needed, and measures to prevent aerosolization from plumbing, as might have occurred in the Amoy Garden outbreak of SARS (*24*), might need to be implemented. Evidence of environmental contamination by SARS-CoV-2 through respiratory droplets and fecal shedding highlights the need for effective decontamination efforts and strict adherence to environmental hygiene, which are pertinent to prevention and control of transmission, including SSEs (*34*).

Behavioral factors include cough hygiene, social customs, health-seeking behavior, and adherence to public health guidance. The risk for SSEs varies widely on the basis of cultural and socioeconomic context. In Sierra Leone, 1 traditional funeral was associated with 28 laboratory-confirmed cases of Ebola (*26**,**35*). Perceptions of risk can influence behavior and the likelihood of SSEs. Underestimation of risk in healthcare facilities resulted in transmission that prolonged the Ebola outbreak in Guinea (*27*). During the MERS outbreak in South Korea, doctor shopping (visiting multiple healthcare facilities after symptoms developed) was associated with SSEs (*36*). For control of COVID-19, behavioral recommendations for the general population to wash hands, cover coughs, and minimize exposing others, as well as rigorous infection control for healthcare workers, are needed.

Response factors include the timely and effective implementation of prevention and control measures within the community and in healthcare settings. These factors can reduce outbreak duration and decrease the reproductive number, thereby reducing the number of persons infected. Because delay of diagnosis is the most common cause of SSEs (*16*), timeliness is critical to prevent or limit their extent (*20*). Rapid identification and isolation of cases will reduce transmission; where necessary, large-scale NPIs should also be implemented in affected areas within 1 week (*37*). Effective case isolation and contact tracing might be sufficient to control a cluster of COVID-19, but the probability of control will decrease with delays in patient isolation from symptom onset (*38*).

## Prevention and Mitigation of SSEs

SSE prevention and mitigation depends, first and foremost, on quickly recognizing and understanding these events. This recognition and understanding enables implementation of control measures specific to the incident and identification of measures, which can reduce the risk for future SSEs. During the SARS epidemic, rapid quarantine and isolation reduced outbreak extent and speed (*19*), and the lack of early detection was the primary cause of a hospital MERS outbreak in South Korea (*39*). An analysis of available data from Hong Kong, Vietnam, Singapore, and Canada found that delaying SARS control measures by just a week could have tripled the size of the epidemic (*7*). A modeling study of control interventions and SSEs in South Korea found that timely interventions (within 1 week), including a government announcement of affected hospitals, reduced the size and duration of MERS transmission (*28*).

Healthcare facilities are critical for prevention and control of SSEs. Targeted control measures include rapid identification and isolation of all potentially infectious patients, including a high index of suspicion for transmissible diseases, and implementation of universal infection control procedures in all areas of all facilities (*20**,**40*). Because individual superspreaders can only be identified retrospectively, universal implementation of triage procedures, rapid diagnosis and isolation, administrative controls (e.g., flow patterns and procedures for patients, visitors, and staff), and engineering controls (e.g., isolation rooms, partitions to protect against respiratory droplets, ventilation systems) are all necessary (*28*). Meticulous infection control is especially needed when performing procedures such as bronchoscopy, intubation, suctioning, sputum induction, and nebulizer therapies, which can enable what would normally be a droplet-transmitted infection to become aerosolized and therefore able to be more widely disseminated. If these types of procedures are needed, they should be performed by using strict infection control procedures and, when possible, in airborne infection isolation units.

SSEs in healthcare settings can be associated with increased illness and death because many infections occur among patients with underlying conditions, which can delay diagnosis and exacerbate pathologic changes (*41*,*42*). Most tuberculosis is spread by patients who have not yet been given a diagnosis, rather than by failure to effectively isolate these patients (*43*). Risk factors for SSEs of SARS among 86 wards in Guangzhou, China, and 38 wards in Hong Kong were related to inadequate infection prevention and control, including insufficient availability of washing and changing facilities for staff, performing resuscitation on the ward, staff working while experiencing symptoms, and use of oxygen therapy or positive pressure ventilation (*25*). One patient in China who had only abdominal symptoms was not initially suspected of having COVID-19 and was admitted to a surgical ward; >10 healthcare workers and >4 patients were presumed to have been infected by this patient (*3*). It is essential that healthcare facilities implement infection control guidelines for COVID-19 rigorously. It is also essential that any nosocomial transmission is analyzed to identify the modes of spread, which will inform best strategies for prevention.

SSEs also occur in settings other than healthcare settings (*44*). The SARS outbreak in Hong Kong was characterized by 2 SSEs responsible for >400 infections (*45*); 1 guest at the Metropole Hotel was the index case for 4 national and international clusters (*46*). Community-wide NPIs, including risk communication to the public on social distancing, hand and respiratory hygiene, and criteria for either self-isolation or safer presentation to the hospital, can limit community transmission. During the SARS outbreak, effective communication appears to have reduced time from symptom onset to hospital admission and decreased the number of persons with whom patients had contact before isolation (*25*). The combination of facility-based and population-based interventions ended SARS transmission (*19**,**47*).

A study modeling the impact of interventions in Wuhan found that, although early identification and isolation reduced the number of infections somewhat, integrated implementation of NPIs decreased the number of cases rapidly and substantially and drove the reproductive number to <1 (C. Wang et al., unpub. data, https://doi.org/10.1101/2020.03.03.20030593). If NPIs had been implemented 2 weeks earlier, an estimated 86% of cases might have been prevented (*48*). For COVID-19, broad infection prevention and control measures include cough and hand hygiene, self-isolation by staying home if sick, and avoiding infection during care-seeking and caregiving.

## Conclusions

COVID-19 has already killed more persons than SARS and MERS combined. Both of these coronavirus infections were fueled by SSEs. Understanding transmission dynamics associated with SSEs and their control during other coronavirus outbreaks can help inform current public health approaches to SARS-CoV-2. Anticipated heterogeneity in transmission should be used to plan disease control programs and risk-stratify populations for public health interventions. Countries should develop and implement protocols for implementation of rapid identification, diagnosis, and isolation of patients; effective infection prevention and control practices in healthcare facilities; and timely and relevant risk communication. Such measures can mitigate the impact of SSEs, which have been major drivers of recent epidemics.

Because delay in diagnosis and failure to rapidly implement isolation and response measures have fueled previous SSEs, countries should have plans and operational capacities in place during the containment phase of the response for immediate investigation and implementation of control measures. During the later mitigation phase, when surveillance and laboratory resources are limited, surveillance and focused response efforts should prioritize environments and settings at high risk for SSEs, including closed environments such as healthcare facilities, nursing homes, prisons, homeless shelters, schools, and sites of mass gatherings while community-wide NPIs are implemented more broadly.

Targeted and rapidly implemented public health interventions to prevent and mitigate SSEs are critical for early interruption of transmission during the containment phase and to reduce the effect on the disruption of healthcare services and society during the mitigation phase. Because of the societal and cultural underpinnings of behavioral and environmental factors including the local acceptability of adoption of NPIs, early engagement of communities, including an in-depth understanding of knowledge, attitudes, and practices relevant to the pandemic will be critical to response efforts during all phases.
